# Azithromycin in viral infections

**DOI:** 10.1002/rmv.2163

**Published:** 2020-09-23

**Authors:** Madeleine E. Oliver, Timothy S. C. Hinks

**Affiliations:** ^1^ New College, University of Oxford Oxford UK; ^2^ Nuffield Department of Medicine Experimental Medicine, Respiratory Medicine Unit and National Institute for Health Research (NIHR), Oxford Biomedical Research Centre (BRC) University of Oxford Oxford UK

**Keywords:** azithromycin, coronavirus, COVID‐19, macrolide, mechanism, review, SARS‐CoV‐2, virus

## Abstract

Azithromycin (AZM) is a synthetic macrolide antibiotic effective against a broad range of bacterial and mycobacterial infections. Due to an additional range of anti‐viral and anti‐inflammatory properties, it has been given to patients with the coronaviruses SARS‐CoV or MERS‐CoV. It is now being investigated as a potential candidate treatment for SARS‐CoV‐2 having been identified as a candidate therapeutic for this virus by both in vitro and in silico drug screens. To date there are no randomised trial data on its use in any novel coronavirus infection, although a large number of trials are currently in progress. In this review, we summarise data from in vitro, murine and human clinical studies on the anti‐viral and anti‐inflammatory properties of macrolides, particularly AZM. AZM reduces in vitro replication of several classes of viruses including rhinovirus, influenza A, Zika virus, Ebola, enteroviruses and coronaviruses, via several mechanisms. AZM enhances expression of anti‐viral pattern recognition receptors and induction of anti‐viral type I and III interferon responses. Of relevance to severe coronavirus‐19 disease (COVID‐19), which is characterised by an over‐exuberant innate inflammatory response, AZM also has anti‐inflammatory properties including suppression of IL‐1beta, IL‐2, TNF and GM‐CSF. AZM inhibits T cells by inhibiting calcineurin signalling, mammalian target of rapamycin activity and NFκB activation. AZM particularly targets granulocytes where it concentrates markedly in lysosomes, particularly affecting accumulation, adhesion, degranulation and apoptosis of neutrophils. Given its proven safety, affordability and global availability, tempered by significant concerns about antimicrobial stewardship, there is an urgent mandate to perform well‐designed and conducted randomised clinical trials.

**Abbreviations**
AZM: azithromycin
CAP: community acquired pneumonia
CCL: C‐C motif ligand
CD: cluster of differentiation
CF: cystic fibrosis
COVID‐19: coronavirus‐19 disease
COX: cyclooxygenase
cPLA2: cytosolic phospholipase A2
CRP: C‐reactive protein
CXCL: C‐X‐C motif ligand
DPB: diffuse panbronchiolitis
ERK: extracellular signal‐regulated kinase
GM‐CSF: granulocyte‐macrophage colony‐stimulating factor (CSF2)
HCQ: hydroxychloroquine
hPSC: human pluripotent stem cell
ICU: intensive care unit
IL: interleukin
IFN: interferon
IRF3: Interferon Regulatory Factor 3
ISG: interferon‐stimulated gene
IVM: ivermectin
LMWH: low molecular weight heparin
MAPK: mitogen‐activated protein kinase
MCL1: myeloid cell leukaemia sequence 1
MDA5: melanoma differentiation‐associated protein 5
MxA: myxoma virus resistance A
NALP3: NACHT, LRR, and PYD domains‐containing protein 3
NCT: National Clinical Trial
PBEC: primary bronchial epithelial cell
PGE2: prostaglandin E2
PRR: pattern recognition receptor
RIG‐1: retinoic acid‐inducible gene 1
RV: rhinovirus
SARS: severe acute respiratory syndrome
TBK1: TANK‐binding kinase 1
TGF‐beta: transforming growth factor beta
TLR: Toll‐like receptor

## 1 INTRODUCTION

Azithromycin (AZM) is a second‐generation, broad‐spectrum, synthetic macrolide antibiotic used since the early 1980s^1^, ^2^ to treat a wide range of bacterial and mycobacterial infections of respiratory and skin infections. It is therefore on the WHO list of essential medications,^3^ and manufactured on a large scale globally. Its antibacterial activity derives from its ability to bind to the 50S ribosomal subunit, inhibiting protein synthesis.^4^ It also has an intriguing range of anti‐viral and anti‐inflammatory properties, and is now being investigated as a potential candidate treatment for viruses including SARS‐CoV‐2, which causes coronavirus‐19 disease (COVID‐19). It has been used as a treatment in previous coronavirus diseases during the epidemics of severe acute respiratory syndrome (SARS) in 2003 and Middle East respiratory syndrome (MERS)^5^ in 2012, but to date there are no randomised trial data on its use in any novel coronavirus infection. Its proven safety, affordability and global availability make it an attractive candidate for repurposing as a treatment for COVID‐19. Given the expected massive global impact of COVID‐19, particularly in low‐to‐middle income countries, it is important not only to develop therapies that treat the virus successfully, but also to ensure that these therapies are readily implementable at all levels of development and economy.^6^ This review summarizes the current understanding of the anti‐viral and anti‐inflammatory effects of AZM, with a view to supporting our knowledge in the pursuit of a COVID‐19 therapy that can help tackle this virus globally.

## 2 MECHANISMS OF ANTI‐VIRAL EFFECTS

A range of human in vitro and in vivo studies provide evidence of anti‐viral activity of macrolides across a broad range of viral species and families (Table 1). Some studies suggest improved symptom resolution and reduction,^17^, ^18^, ^19^, ^20^, ^21^, ^22^ although not all studies have observed these effects.^23^, ^24^, ^25^, ^26^

**TABLE 1:** Viral infections in which azithromycin has demonstrated anti‐viral effects

Pathogen	Findings	Method	Study
Human rhinovirus (Picornavirus)	Enhanced viral‐induced type I and III IFN leading to reduced RV replication and release	In vitro study. PBEC. 10 μM, 50 μM	Gielen et al^7^
	Reduced RV replication	In vitro study. PBEC from cystic fibrosis patients. 50 μM	Schogler et al^8^
		In vitro study. PBECs and BEAS‐2B cells 50 μM, 10 μM	Porter et al^9^
	Increases RV PRR presentation	In vitro study. PBEC. 10 μM, 50 μM	Gielen et al^7^
	Induces anti‐viral ISGs viperin and MxA	In vitro study. PBECs and BEAS‐2B cells 50 μM, 10 μM	Porter et al^9^
Coronaviruses (alpha and beta)	AZM associated with reduced viral load in children with coronaviruses	Clinical trial. Dose ≥20 mg/kg	Doan et al^10^
Zika (Flavivirus)	AZM markedly reduces viral proliferation and virus‐induced cytopathic effects	In vitro study. U87 glial cells and hPSC‐derived astrocytes, 0 μM to >100 μM	Retallack et al^11^
	AZM upregulates type I and III interferon responses	In vitro study. HT‐29 human colon epithelial cell line and A549 lung epithelial cell line. 10 μM, 50 μM	Li et al^12^
	AZM upregulates viral pathogen recognition receptors MDA5 and RIG‐1	In vitro study. A549 lung epithelial cell line. 10 μM, 50 μM	
	AZM increases levels of phosphorylated TBK1 and IRF3	In vitro study. HT‐29 human colon epithelial cell line, 10 μM, 50 μM	
		Human primary fibroblasts, 5 μM, 20 μM. RAW264.7 macrophage cells 1.5 μM, 3 μM	
Enteroviruses (Picornaviruses)	AZM improved survival and clinical symptom scores in murine model	In vivo study. Mice infected i.p. with EV‐A71‐MZ‐MA1. AZM dose 30 mg/kg/day	Zeng et al^13^
Ebola (Ebola viruses)	AZM demonstrates high in vitro anti‐viral potency and low cytotoxicity	In vitro study. HeLa cells (viral replication). HEK 293T cells (viral entry and cytotoxicity). 0.5 to 50 μM	Madrid et al^14^
SARS (Coronavirus)	AZM associated with improvement in 90 d survival rate and time to discontinuation of mechanical ventilation	Single‐centre, retrospective cohort evaluation of hospitalized patients with moderate or severe ARDS, using a propensity score analysis	Kawamura et al^15^
Influenza A (Orthomyxovirus)	Reduction in IL‐6, IL‐8, IL‐17, CXCL9, sTNF and CRP	Randomised, open‐label, multicentre trial of patients with severe influenza. 500 mg AZM od + 75 mg oseltamivir bd/75 mg oseltamivir bd.	Lee et al^16^

Abbreviations: AZM, azithromycin; CRP, C‐reactive protein; CXCL, C‐X‐C motif ligand; hPSC, human pluripotent stem cell; IL, interleukin; IFN, interferon; IRF3, Interferon Regulatory Factor 3; ISG, interferon‐stimulated gene; MDA5, melanoma differentiation‐associated protein 5; MxA, myxoma virus resistance A; PBEC, primary bronchial epithelial cell; PRR, pattern recognition receptor; RIG‐1, retinoic acid‐inducible gene 1, RV, rhinovirus; SARS, severe acute respiratory syndrome; TBK1, TANK‐binding kinase 1.

### 2.1 Mechanisms of anti‐viral effects against rhinovirus

In several clinical trials, macrolides reduced exacerbations in airways diseases, particularly asthma.^27^, ^28^, ^29^, ^30^ As the majority of such exacerbations are triggered by viral infections,^31^ most commonly rhinoviruses (RV),^32^ the effects of macrolides have been studied most extensively against RV. AZM reduces RV replication and release during in vitro infection of primary human bronchial epithelial cells (PBEC).^7^ This finding was replicated in PBEC from patients with cystic fibrosis or healthy controls, where AZM treatment again led to a sevenfold to ninefold reduction in viral shedding, respectively.^8^ The use of AZM alone increased viral‐induced interferons (IFNs) and interferon‐stimulated gene (ISG) mRNA expression and hence production of these gene products.^7^, ^8^ In the latter study, while viral replication was suppressed, AZM did not suppress pro‐inflammatory responses.

In vivo data from the AMAZES study, the largest clinical trial of a long‐term macrolide in airways disease, showed a striking 40% reduction in asthma exacerbations with AZM.^29^ The mechanism is unknown, and would be consistent with an anti‐viral effect, although metagenomic analyses suggest an antibacterial effect reducing *Haemophilus influenzae* ^33^, ^34^ may be the predominant mechanism. The effect on viruses may relate to *H. influenzae* upregulation of ICAM‐1, a major receptor for both *Haemophilus* and rhinovirus (RV).^35^

Other macrolides also have anti‐viral effects in RV infection including Mac5, an oleandomycin macrolide. Both AZM and Mac5 suppressed RV replication and enhanced RV‐induced type I and type III IFNs, as well as the ISGs viperin/MxA.^9^ In this study, macrolides did not affect interleukin (IL)‐6 and ‐8, but secretion of IL‐1β, IL‐6 and IL‐8 were reduced by clarithromycin (another macrolide) in a separate study of RV,^36^ alongside inhibition of viral replication and ICAM‐1. Macrolides such as AZM augment infection‐induced IFN responses.^9^ This is of relevance to coronaviruses as type I IFN inhibit replication of both SARS‐CoV^37^ and SARS‐CoV‐2^38^ in vitro.

RV replication was also inhibited by the macrolides erythromycin^39^ and bafilomycin^40^ in PBEC. In both studies, macrolides reduced RV‐induced NFκB activation and decreased acidity of endosomes in epithelial cells. Bafilomycin inhibited cytokine production and ICAM‐1 expression.

### 2.2 Mechanism of effects in influenza A

In a randomised trial in patients with influenza A receiving oseltamivir, 5 days' adjunctive AZM 500 mg daily was associated with more rapid reductions in plasma concentrations of IL‐6, IL‐8, IL‐17, CXCL9, soluble tissue necrosis factor (TNF) and C‐reactive protein (CRP).^16^ However, this was an open‐label study, with a small sample size (n = 50), and the effect was small, with no significant changes in viral clearance or time to symptom resolution. In a second, larger, open‐label, randomised controlled trial 2 days of clarithromycin 500 mg and naproxen 200 mg twice daily reduced 30 day mortality, high dependency unit admission and hospital stay in 217 elderly patients with H2N2 influenza.^18^ The effect size was marked, although the study is limited by lack of blinding and by the likelihood that much of the effect might be attributable to the antibacterial properties of clarithromycin, as bacterial pneumonias are responsible for a high proportion of influenza deaths, particularly in the elderly.

Nonetheless in vitro clarithromycin reduced viral replication in the A549 human lung cell line.^41^ Likewise clarithromycin reduced viral titres and supernatant cytokines on cultured human tracheal epithelial cells, associated with reduction in surface expression of the influenza A receptor Sα2, 6Gal, inhibition of NFκB and reduced acidification of the endosome required for intracellular release of viral RNA.^42^ More recent data also showed a reduction in H1N1 viral replication in A549 cells with AZM with an IC_50_ of 68 μM, with an effect most apparent during viral particle internalisation.^43^

Some murine studies have investigated macrolides in vivo. Erythromycin improved survival during severe H2N2 infection,^44^ associated with reduced bronchoalveolar lavage (BAL) IFN‐γ, inflammatory cells and nitric‐oxide‐derived free radicals. Other macrolides leucomycin A3, spiramycin and a non‐antibacterial erythromycin derivative (EM900) each reduced weight loss, improved survival and reduced viral protein expression in H1N1 influenza.^45^ In a short‐term H1N1 infection model, AZM reduced expression of viral proteins 2 days post infection.^43^ However, the effect was not sustained, and not associated with a change in virus‐induced weight loss, a sensitive measurement of influenza pathology. Another study found AZM reduced lung viral titres at day 6 post infection, though the effects were not additive to that achieved with oseltamivir in terms of survival, viral titres or cytokine levels,^46^ and so these data remain conflicting.^47^ In a separate influenza study, AZM decreased total leukocyte accumulation in lung tissue and BAL, with the largest reduction being in neutrophils, and associated with decreased inflammatory mediators.

### 2.3 Mechanism of anti‐viral effect in Zika virus

In a drug screen of 2177 compounds against the flavivirus Zika, AZM reduced viral proliferation and virus‐induced cytopathic effects in glial cell lines and human astrocytes.^11^ A further in vitro study found AZM to effectively suppress Zika infection by targeting a late stage in the viral life cycle.^12^ AZM also upregulated expression of type I and III IFNs and several of their downstream ISGs, paralleling activities of AZM in RV.^7^, ^8^ Furthermore, AZM induced enhanced expression of the anti‐viral pattern recognition receptors (PRRs) MDA5 and RIG‐1, as well as the levels of phosphorylated TBK1 and IRF3.

### 2.4 Anti‐viral effects in Ebola

AZM was similarly evaluated in a drug screen for its efficacy as a therapy for Ebola.^14^ While AZM demonstrated high in vitro potency (50% effective concentration [EC_50_] = 5.1 μM) and low toxicity, when tested in an in vivo mouse model it did not consistently improve survival in mice or guinea pigs.

### 2.5 Anti‐viral effects in enteroviruses

Enterovirus A71 (EV‐A71) causes hand, foot and mouth disease in young children. AZM and spiramycin (another macrolide) provided significant in vivo protection against EV‐A71 infection in mice.^13^ Spiramycin impaired EV‐A71 viral RNA synthesis, and it is likely spiramycin and AZM work through a common mechanism, after viral entry, impairing viral RNA synthesis either directly or indirectly.

### 2.6 Anti‐viral effects of AZM in coronaviruses

Human coronaviruses are enveloped positive‐stranded RNA viruses of the *Coronaviridae* family in the *Nidovirales* order comprising four genera (*Alpha‐*, *Beta‐*, *Gamma*‐, *Delta*‐*coronaviruses*).^48^ These viruses are endemic respiratory and gastrointestinal viruses and the *Betacoronavirus* genus includes the pandemic viruses MERS‐CoV, SARS‐CoV and SARS‐CoV‐2. AZM was used in a third of patients treated for MERS‐CoV, although without a clinical evidence base.^5^ A retrospective cohort analysis of 349 patients across 14 sites in Saudi Arabia found no significant reduction in 90‐day mortality (odds ratio [OR] 0.84 95% confidence interval [CI] 0.47‐1.51) or improvement in MERS‐CoV RNA clearance (hazard ratio HR 0.88 [0.47‐1.64] with macrolide use).^5^ However, this was a non‐randomised, retrospective observational study, in which it was unknown on what basis treatment allocation decisions were made, and randomised data are needed.

Interesting data have recently emerged from a mass eradication programme amongst preschool children in Niger. Children up to age 5 were cluster‐randomised by community to a single oral dose of AZM or placebo every 6 months and nasopharyngeal swabs were taken for viral RNA sequencing. After 24 months, AZM use was associated with an eightfold reduction in viral load of *Alphacoronavirus* and a 14‐fold reduction in *Betacoronavirus* viral load, though there was no difference in the prevalence of these viruses.^10^

Since the outbreak of the current SARS‐CoV‐2 pandemic, several drug screens have investigated potential candidate drugs against this virus. A screen of 1520 approved and off‐patent drugs identified 90 drugs which inhibited SARS‐CoV‐2 viral replication at 10 μM.^48^ These included ATPase proton pump inhibitors, protease inhibitors, viral protease inhibitors, drugs targeting the angiotensin pathway and AZM. AZM had an EC_50_ of 2.12 μM and EC_90_ 8.65, and selectivity index >19, which is very comparable to the control compound remdesivir (EC_50_ = 1.65, EC_90_ = 2.52), the only anti‐viral with proven clinical efficacy against SARS‐CoV‐2 in clinical trials to date.^49^, ^50^ Likewise AZM was also identified as a target in a bioinformatic screening analysis of potentially relevant pathways with the potential for development into pharmaceutically acceptable forms,^51^ in this case by inhibiting autophagy via inhibition of the vacuolar ATPase necessary for autophagosome‐lysosome fusion.^52^ A focussed study on two candidate molecules, hydroxychloroquine and AZM, suggested a synergistic inhibition of SARS‐CoV‐2 replication in Vero cells at 5 and 10 μM concentrations, respectively.^53^ This synergy has been proposed to allow effective use of hydroxychloroquine at less toxic concentrations, and is an approach tried in a small observational study which suggested enhanced virological clearance with hydroxychloroquine, particularly in combination with AZM.^54^ However this study was very small, with AZM data from only six patients, and was open‐label and non‐randomised, allowing no useful conclusions to be drawn. Moreover, there are concerns that combination therapy may enhance cardiovascular side effects as both molecules individually can cause prolongation of the QT interval.^55^ This combination has been tested in non‐human primates, where a significant anti‐viral effect was not seen in the five macaques which received AZM in addition to hydroxychloroquine.^56^

## 3 ANTI‐INFLAMMATORY EFFECTS

**FIGURE 1:**
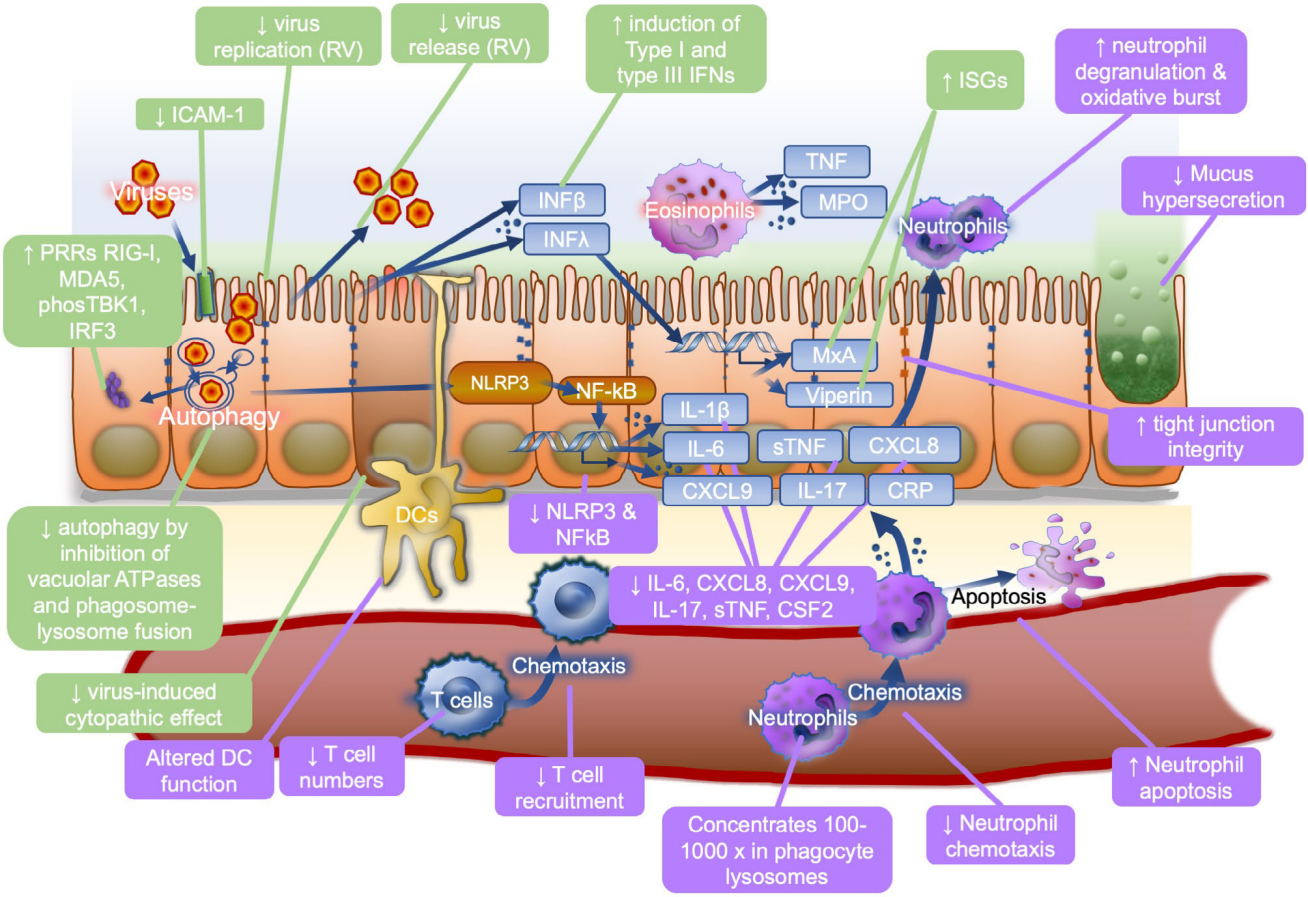
Anti‐viral and anti‐inflammatory effects of macrolides. Schematic showing major proposed mechanisms of azithromycin anti‐viral (green) and anti‐inflammatory or immunomodulatory (purple) activities. AZM, azithromycin; CRP, C‐reactive protein; CSF2, colony‐stimulating factor 2 (GM‐CSF); CXCL, C‐X‐C motif chemokine ligand; DC, dendritic cell; ICAM1, intracellular cell adhesion molecule 1; IFN, interferon; IL, interleukin; IRF3, Interferon Regulatory Factor 3; ISG, interferon‐stimulated gene; MDA5, melanoma differentiation‐associated protein 5; MPO, myeloperoxidase; MxA, myxoma resistance protein 1; NFκB, nuclear factor kappa B; NLRP3, nucleotide‐binding oligomerisation domain; phosTBK1, phosphorylated TANK‐binding kinase 1; PRR, pattern recognition receptor; RIG‐I, retinoic acid‐inducible gene I; RV, rhinovirus; Th17, type‐17 T‐helper; TLR, Toll‐like receptor; (s)TNF, (soluble) tissue necrosis factor

Whilst viruses can cause tissue damage by direct cytopathic effects on the infected cells, morbidity and mortality in severe disease are typically attributable to the host inflammatory response, including in COVID‐19.^57^ AZM and other macrolides have a number of immunomodulatory properties which have proven clinical efficacy in a broad range of respiratory diseases including asthma,^29^ COPD,^58^ post lung transplant obliterative bronchiolitis^59^ and diffuse pan bronchiolitis (DPB).^60^, ^61^, ^62^, ^63^ In DPB, a dramatic increase in survival^60^, ^62^, ^63^ has been attributed to the ability of AZM to inhibit dysregulated IL‐1β, IL‐2, TNF and GM‐CSF.^64^ Therefore, the anti‐inflammatory properties of AZM (summarised in Table 2 and Figure 1) may be clinically important in the management of viral diseases.

**TABLE 2:** Immunomodulatory and anti‐inflammatory properties of azithromycin

Property	Effect	Specific Findings	Study
General anti‐inflammatory properties			
Destabilisation of NALP3 mRNA levels	Decreased IL‐beta production	LPS‐stimulated THP‐1 monocytes. AZM reduced IL‐1beta, NALP3 protein and NFκB activity	Lendermon et al^65^
Inhibition of inflammatory cytokine release	Decreased CXCL8 (IL‐8), NFκB and AP‐1 from epithelial cells	Clinical trial in recurrent genital *C. trachomatis* infection. Decreased IL‐1beta, CXCL‐1, ‐5, ‐8, ‐9, CCL2, ‐5, MCL1, MAPK1	Srivastava et al^66^
		Airway epithelial cell lines. Decrease in CXCL8 mRNA, and NFκB and AP‐1 binding	Cigana et al^67^
	Decreased CXCL8 (IL‐8), MAPK and 8‐isoprostane in airway smooth muscle cells	IL‐17‐stimulated primary human airway smooth muscle cells	Vanaudenaerde et al^68^
	Decreased PGE2 synthesis	Human polymorphonuclear and mononuclear leukocytes. Decreased LPS‐induced PGE2 by suppression of cPLA2, COX‐1, COX‐2	Miyazaki et al^69^
	Decreased TNF from cystic fibrosis airway epithelial cells	Human CF and non‐CF cell lines. Decreased TNF mRNA and protein and NFκB and Sp1 binding	Cigana et al^70^
	Decreased GM‐CSF	Airway epithelial (A549) cell lines. Reduced TNF‐induced GM‐CSF mRNA and protein expression	Yamasawa et al^71^
	Reduction of cytokine‐induced endothelin 1 expression in epithelial cells	Human bronchial epithelial cells. Erythromycin and clarithromycin reduced enfothelin‐1 expression	Takizawa et al^72^
Inhibition of endocytosis/induction of phospholipidosis			
	Decreases motility and fluidity of the plasma membrane	J774 macrophage cell line	Tyteca et al^73^
	Slows membrane trafficking towards lysosomes	J774 macrophage cell line	Tyteca et al^74^
	Inhibition of fluid phase endocytosis of macromolecules	J774 macrophage cell line	Tyteca et al^74^
	Down‐regulates and delays recycling of surface transferrin receptors	J774 macrophage cell line	Tyteca et al^74^
	Inhibition of pinocytosis of macromolecules and their transport from plasma membrane to endo/lysosomes	J774 macrophage cell line	Tyteca et al^74^
	Increase of lysosomal hydrolase activity in fibroblasts	Fibroblast homogenates. Increased activity of sulfatase A, phospholipase A1, cathepsin B	Gerbaux et al^75^
	Lysosomal enzyme depletion/extracellular secretion of lysosomal enzymes	Rat kidney cells. Redistribution of mannose 6‐phosphate receptor	Ikeda et al^76^
Effects on airway inflammatory cells			
Accumulation intracellularly within phagocytes	Prolonged macrolide retention intracellularly	Human in vivo 210 h T1/2 in neutrophils. Concentration in alveolar macrophages, in neutrophils, in phagocytic and epithelial cell lines	Wildfeuer et al, Capitano et al, Bosnar et al^77^, ^78^, ^79^
	Prolonged AZM retention within neutrophils	Concentrations 2000 to 3000 times higher in neutrophils than plasma	Wilms et al^80^
	Accumulation of macrolides in alveolar macrophages	Human in vivo 500‐fold accumulation in alveolar macrophages	Lucchi et al, Capitano et al^77^, ^81^
Neutrophils	Inhibition of neutrophil chemotaxis	Murine pseudomonas model and human neutrophils. Reduced neutrophil chemotaxis via ERK‐1 and ERK‐2	Tsai et al^82^
	Down regulation of neutrophil chemokine production	Human blood. Decreased azurophilic granule enzyme activities	Culić et al, Tsai et al^83^, ^84^
	Attenuation of neutrophil oxidative burst	Human blood neutrophils	Nozoe et al^85^
	Down regulation of MPO production	Human in vivo blood neutrophils. Decreased MPO concentration	Culić et al^84^
	Increased neutrophil apoptosis	Human in vivo blood neutrophils. Increased neutrophil apoptosis 28 days post dose	Culić et al^84^
	Inhibition of neutrophil elastase and MMP9	Human in vivo. Clarithromycin reduced airway neutrophil elastase and MMP9	Simpson et al^86^
Macrophages	Increased phagocytosis	Human alveolar macrophages. Increased phagocytosis of apoptotic bronchial epithelial cells and neutrophils	Hodge et al, Yamaryo et al^87^, ^88^
	Macrophage lysosomes more resistant to oxidant challenge	Human alveolar macrophages ex vivo. Reduced oxidative lysosomal membrane permeabilisation	Persson et al^89^
	Polarization towards M2 phenotype	In vitro polarised J774 macrophage cell line. Increased M2 markers mannose receptor, CD23, arginase, decreased CCR7	Murphy et al^90^
	Reduction in production of GM‐CSF and IL‐1beta	Murine LPS challenge. Decreased GM‐CSF, IL‐1beta, TNF, CCL2	Bosnar et al^91^
	Suppression of IL‐12p40 by macrophages	LPS‐stimulated macrophage cell lines. Decreased IL‐12p40 induction by inhibited AP‐1, NFAT, ICSBP binding	Yamauchi et al^92^
	Increased mannose receptor expression	Human in vivo trial. Increased mannose receptor expression and phagocytosis	Hodge et al^93^
	Decreased CXCL8 (IL‐8) production	Human ex vivo blood and lung macrophages. CXCL8 inhibited at 400 mg/L	Kurdowska et al^94^
Dendritic cells	Modulation towards a regulatory phenotype	Monocyte‐derived dendritic cells enhanced IL‐10 release and inhibited IL‐6, IL‐12p40, CXCL10, CXCL11 and CCL22 release	Polancec et al, Sugiyama et al^95^, ^96^
	CD40, CD86, and MHCII expression inhibited	Murine bone marrow derived DCs and murine histoincompatible bone marrow transplant model. Decreased CD40 and CD86	Iwamoto et al^97^, ^98^
Natural Killer cells	Inhibition of cytotoxic function through down regulation of perforin expression	Human NK cells. Decreased CD69, perforin and cytotoxicity	Lin et al^99^
Effects on airway mucosal stromal cells			
Smooth muscle cells	Antiproliferative effect	Rabbit tracheal smooth muscle cells. Reduced proliferation, increased autophagy	Stamatiou et al^100^
	Relaxant effect	Rabbit tracheal smooth muscle cells. Smooth muscle relaxation	Daenas et al^101^
Airway epithelium			
	Enhanced airway epithelial integrity	Increased transepithelial electrical resistance by altered processing of tight junction proteins	Asgrimsson et al, Halldorsson et al^102^, ^103^
	Inhibition of inflammatory mucin release	Human cell lines and primary cells. Inhibition of MUC5AC production	Imamura et al, Ribeiro et al^104^, ^105^
	Modulated CXCL8 (IL‐8) production	Human bronchial epithelial cells. Increased CXCL8 release	Shinkai et al^106^
	Reduced CXCL8 (IL‐8)	Human trial. Roxithromycin reduced CXCL8 in nasal lavage in chronic rhinosinusitis, with clinical improvement	Wallwork et al, Yamada et al^107^, ^108^

Abbreviations: CCL, C‐C motif ligand; CD, cluster of differentiation; CF, cystic fibrosis; CXCL, C‐X‐C motif ligand; DPB, diffuse panbronchiolitis; COX, cyclooxygenase; cPLA2, cytosolic phospholipase A2; ERK, extracellular signal‐regulated kinase; GM‐CSF, granulocyte‐macrophage colony‐stimulating factor (CSF2); IL, interleukin; MAPK, mitogen‐activated protein kinase; MCL1, myeloid cell leukaemia sequence 1; NALP3, NACHT, LRR, and PYD domains‐containing protein 3; PGE2, Prostaglandin E2; TGF‐beta, transforming growth factor beta.

### 3.1 Immunomodulatory effects on phagocytes

AZM is rapidly absorbed after oral administration with a large volume of distribution^109^ and a long serum half‐life of approximately 3 days,^110^ leading to a high and sustained tissue concentration. A striking feature of macrolides is that they can accumulate in host cells including epithelial cells and most particularly in phagocytes where they may concentrate 100‐ to 3000‐fold in the lysosomes of phagocytes,^77^, ^81^, ^111^, ^112^ being subsequently retained intracellularly^78^, ^80^, ^81^ and released when these cells die. Therefore typical AZM concentrations after one‐three 500 mg oral doses may be 0.29 μM (0.22 mg/L) in plasma, but 12 μM in lung tissue homogenate, 48 μM in bronchial washings and 260 μM in alveolar macrophages.^113^ Several studies have observed initial stimulatory effects of AZM on immune and epithelial cells. Acutely, AZM stimulates neutrophil degranulation and phagocytosis‐associated oxidative burst, mediated via modulation of Erk1/2 signalling.^79^ These initial stimulatory effects are followed by modulation of transcription factors activator protein (AP)‐1, nuclear factor kappa B (NFκB), inflammatory cytokine and mucin release, with overall anti‐inflammatory effects.

Many inflammatory cytokine levels are reduced by AZM, including IL‐6, IL‐8 (CXCL8), TNF^114^ and GM‐CSF, as well as matrix metalloproteases MMP‐1, ‐2, ‐9, ‐10 and ‐13, and modulation of lipid metabolism and cell cycle pathways (Table 2).^70^

One pathway for macrolide immunomodulation is through binding to macrophilin‐12 inhibiting calcineurin and thus T cell activation, via the same mechanism as tacrolimus,^29^ with consequent downstream inhibition of many immune cells including eosinophils and basophils.^68^ Macrolides also inhibit mammalian target of rapamycin (mTOR) activity, also important in T cell activation and granulocyte differentiation,^115^ suppressing cell proliferation and CD4 + T cell cytokine secretion.^116^ A third pathway modulated by macrolides is activity of the transcription factors NFκB and AP‐1. AZM suppresses p65, a component of NFκB^117^ and attenuates NFκB activation in lung epithelial cells.^118^ This inhibition reduces epithelial cell IL‐8 production,^67^, ^118^ stromal cell proliferation^66^ and macrophage expression of IL‐12p40^119^ and, indirectly, IL‐1β.^65^, ^92^

In macrophages, AZM has several effects including attenuation of lipopolysaccharide‐induced pro‐inflammatory cytokines through inhibition of AP‐1, and increasing phagocytosis,^120^ enhancing the resistance of lysosomes to oxidant challenge^93^ and promoting M2 polarization of macrophages.^89^, ^90^, ^119^ Macrolides including AZM can also increase the phagocytosis of apoptotic epithelial cells^121^ and neutrophils by macrophages,^87^ which can ameliorate inflammation.

### 3.2 Effects on other cell types

In vitro AZM modulated differentiation and maturation of dendritic cells towards a regulatory phenotype with increased phagocytic capacity,^88^, ^95^ with inhibited expression of CD40, CD86, MHCII and IL‐12.^96^, ^97^ Likewise AZM inhibited the cytotoxic function of natural killer cells through down regulation of perforin.^98^

AZM may have anti‐inflammatory effects directly on epithelial cells, such as suppression of GM‐CSF release,^99^ TNF,^114^ inhibition of IL‐8 production^118^ and modulation of the anti‐viral PRRs RIG‐I and MDA5.^7^ AZM inhibition of AP‐1 activation reduces production of MUCA5C responsible for inflammation‐induced changes in airway mucus.^71^, ^122^ Macrolides inhibit airway epithelial cell mucus secretion^123^ and directly inhibit neutrophil elastase.^124^, ^125^ Another effect of macrolides on airway epithelial cells observed in vitro is increased epithelial barrier integrity by alterations in tight junction proteins, including claudins.^102^, ^126^

Overall, macrolides have a number of inhibitory effects on the production of pro‐inflammatory cytokines from innate and adaptive immune cells, and most markedly on the accumulation, adhesion and apoptosis of pulmonary neutrophils.

## 4 POTENTIAL CLINICAL UTILITY IN COVID‐19

Beyond its anti‐viral properties, the anti‐inflammatory effects of AZM may be clinically important in treating the cytokine storm which is a prominent feature of influenza A and of COVID‐19. An exuberant production of pro‐inflammatory cytokines including TNF, IL 1β, IL‐6, G‐CSF and IP‐10 are significantly increased in COVID‐19 disease,^103^ and are associated with features of hemophagocytic lymphohistiocytosis^127^ and interstitial mononuclear inflammatory infiltrates, dominated by lymphocytes,^128^ and with poor clinical outcomes.^103^ However, in contrast to influenza A, where this cytokine storm occurs early in disease, most COVID‐19 related deaths occur due to sudden, late respiratory failure, on average at day 14 after symptom onset,^129^ by which point viral loads have markedly decreased. Severe COVID‐19 disease is associated with loss of alveolar macrophages^130^ and an influx of pro‐inflammatory monocyte‐derived macrophages.^131^

The importance of controlling this inflammation is demonstrated by the recent positive findings of the RECOVERY trial showing a significant mortality benefit with dexamethasone in patients with severe COVID‐19 disease and respiratory failure.^132^ Interestingly, there was no benefit in those randomised at earlier disease stages, consistent with a lower degree of inflammation in these individuals, and suggesting other anti‐inflammatory approaches with fewer side effects might be valuable. The lag between symptom onset and severe disease provides a therapeutic window in which AZM anti‐inflammatory properties may reduce severe pulmonary inflammation, benefiting from the propensity of macrolides to accumulate in phagocytes,^60^, ^111^ which targets them specifically to the sites of pathology in COVID‐19.

It is understandable therefore, that more than 80 clinical trials have been designed to test AZM efficacy in COVID‐19 (Table S1). These differ significantly from each other according to dosing regime, duration of therapy, whether being used in combination with hydroxychloroquine and, critically, according to the population being studied. Those recruiting in primary care will tend to study the anti‐viral effects in early disease, whilst those recruiting in secondary care will be studying more the anti‐inflammatory effects important in late disease. The first trial to publish results compared standard care with hydroxychloroquine (HCQ) 400 mg twice daily or with HQC 400 mg twice daily and AZM 500 mg once daily for 7 days in hospitalised patients with a median duration of symptoms of 7 days prior to randomisation.^133^ There was no reduction in symptoms or requirement for ventilation with either HCQ plus AZM compared with HCQ alone (odds ratio 0.82; 95% confidence interval 0.47‐1.43), but data from other populations, disease stages and without HCQ are urgently needed. If studies show clinical efficacy it will be essential to determine which populations benefit and what criteria to use as clinical indications for therapy. There is also a need for further human in vivo mechanistic studies to determine which of the manifold potential mechanisms are dominant in patients with disease.

AZM is generally well tolerated, the most common side effect being diarrhoea,^29^ it is contraindicated in pregnancy and known hypersensitivity. Whilst there have been concerns about cardiovascular risk, huge epidemiological studies suggest these are very small effects (eg, 47 extra deaths/million prescriptions) or perhaps no effect when corrected for confounding,^134^ and a Cochrane review of 183 trials found no evidence of an increase in cardiac disorders with macrolides (OR 0.87).^135^ Concerns have been raised about the potential interactions between HCQ and AZM increasing risk of side effects. It should be used in caution in those receiving some other drugs including fluoroquinolones such as moxifloxacin and levofloxacin, and in patients with ongoing proarrhythmic conditions, and QT prolongation was more frequent in people with COVID‐19 receiving HCQ (14.6%) or the combination of HCQ and AZM (14.7%) than standard care (1.7%), an effect likely purely attributable to the HCQ.^133^

Given the significant clinical utility of AZM as an antibiotic, the current rapid spread of antimicrobial resistance is of particular concern. Widespread use of AZM to treat viral infections runs an inevitable risk of increasing the development of drug‐resistant bacteria, and indeed there are good data that increasing rates of macrolide resistance in *Streptococcus pneumoniae* in the United States correlated closely with global sales of AZM, while in some regions such as China resistance rates approach 90% for *Mycoplasma pneumoniae* and nearly 100% for *S. pneumoniae*.^110^ Resistance is a particularly high risk with macrolides due to several features including their long half‐life, the widespread use of the drug, and the high‐level macrolide, lincosamide and streptogramin (MLS_B_) resistance phenotype attributable to mutations in the *erm* gene and which are frequently associated with resistance to other classes of antibiotics on the same mobile genetic elements.^110^ Therefore, it will be important to understand the potential anti‐viral and anti‐inflammatory properties of other novel macrolides which have been synthesised but do not have broad‐spectrum antibacterial properties and might therefore reduce development of resistance^136^ and disruption to the natural microbiome.^137^

## 5 CONCLUSIONS

As a therapeutic class, macrolides, and in particular AZM, with its long therapeutic half‐life, good safety profile and very strong evidence base in bacterial diseases are fascinating molecules. Macrolides undoubtedly have broad‐spectrum anti‐viral properties in vitro. AZM consistently emerges as a candidate molecule in anti‐viral drug screens against respiratory viruses, and there are tantalising hints of clinical efficacy in clinical studies to date. The additional anti‐inflammatory properties displayed by some macrolides, including AZM, may well prove to be clinically important in reducing immunopathology in some viral diseases, not least against the pandemic *Betacoronaviruses* in which activation of an over‐exuberant inflammatory cascade seems to be critical to mortality. However there is currently insufficient evidence to justify their use clinically, but rather, a clear mandate to perform well‐designed and conducted randomised trials in patients with chronic airways disorders and those with pandemic respiratory viruses including influenza A, SARS‐CoV‐2 and in future pandemics of novel coronaviruses which increasingly appear to be an inevitable prospect.

## CONFLICT OF INTEREST

M.E.O. None. T.S.C.H. has received unrestricted research grants from the Wellcome Trust (211050/Z/18/z, 211050/Z/18/A) and from the National Institute for Health Research (NIHR) Oxford Biomedical Research Centre (BRC), the University of Oxford COVID‐19 Research Response Fund and from Pfizer to support conduct of a trial of azithromycin in COVID‐19.

## AUTHOR CONTRIBUTIONS

Madeleine E. Oliver and Timothy S. C. Hinks jointly conceived the article, conducted the literature review and drafted the manuscript. All authors approved the final manuscript.

## Supporting information


**Table S1** Current clinical trials of azithromycin in SARS‐CoV‐2

## Data Availability

Data sharing not applicable to this article as no datasets were generated or analysed during the current study.
