# Supplementary material for: Azithromycin in viral infections
Source: Rev Med Virol. 2020 Sep 23;31(2):e2163. doi: 10.1002/rmv.2163 (PMC7536932; doi:10.1002/rmv.2163)
Supplement: Supplementary file 1 — Table S1 Current clinical trials of azithromycin in SARS‐CoV‐2 [file RMV-31-0-s001.docx]

| **Table 3** Current clinical trials of azithromycin in SARS-CoV-2 | | | | | | | | | |
| --- | --- | --- | --- | --- | --- | --- | --- | --- | --- |
|  |  |  |  |  |  |  |  |  |  |
| **Study acronym** | **Study name** | **NCT** | **Recruiting** | **Interventions** | **Location** | **Population** | **Estimated enrolment** | **Date registered** | **Design** |
|  |  |  |  |  |  |  |  |  |  |
| RECOVERY | Randomised Evaluation of COVID-19 Therapy (RECOVERY) | 04381936 | Y | Std care / low dose corticosteroid / HCQ / lopinavir-ritonavir/AZM / convalescent plasma/tocilizumab | Oxford, UK | COVID | 12000 | 11.05.2020 | Randomised |
| REMAP-COVID | Randomized, Embedded, Multifactorial Adaptive Platform Trial for Community- Acquired Pneumonia (REMAP-CAP) | 02735707 | Y | Macrolide (AZM preferred, clarithromycin or roxithromycin may be substituted)/ corticosteroids / lopinavir-ritonavir/ HCQ / lopiavir-ritonavir+HCQ / IFNbeta1a / anakinra / tocilizumab / sarilumab | International | Severe CAP, ICU admission, COVID | 7100 | 13.04.2016 | Randomised |
| ACTION | Azithromycin for COVID-19 Treatment in Outpatients Nationwide (ACTION) | 04332107 | Y | AZM / placebo | California, USA | COVID | 2271 | 02.04.2020 | Randomised |
|  | Evaluating the Efficacy of Hydroxychloroquine and Azithromycin to Prevent Hospitalization or Death in Persons With COVID-19 | 04358068 | Y | AZM/ HCQ | NIAID, USA | Mild COVID | 2000 | 22.04.2020 | Randomised |
| ACT COVID19 | Anti-Coronavirus Therapies to Prevent Progression of Coronavirus Disease 2019 (COVID-19) Trial (ACT COVID19) | 04324463 | Y | AZM + HCQ / Std care / AZM + IFNbeta / IFNbeta | Canada | COVID | 1500 | 27.03.2020 | Randomised |
|  | Efficacy and Safety of Hydroxychloroquine and Favipiravir in the Treatment of Mild to Moderate COVID-19 | 04411433 | Y | Favipiravir / favipiravir + HCQ / favipiravir + AZM / HCQ/ HCQ + AZM | Turkey | Mild COVID | 1000 | 02.06.2020 | Randomised |
|  | Efficacy of Natural Honey Treatment in Patients With Novel Coronavirus | 04323345 | Y | Honey +lopinavir/ritonavir tablets or Arbidol or chloroquine phosphate or Hydroxychloroquine or oseltamivir with or without azithromycin / lopinavir/ritonavir tablets or Arbidol or chloroquine phosphate or Hydroxychloroquine or oseltamivir with or without azithromycin | Egypt | COVID | 1000 | 26.03.2020 | Randomised |
|  | Prevention of SARS-CoV-2 in Hospital Workers s Exposed to the Virus (PREP-COVID) | 04344379 | Y | HCQ / HCQ placebo / AZM | France | Hospital workers, no COVID | 900 | 14.04.2020 | Randomised |
| ATOMIC2 | A Multi-centre Open-label Two-arm Randomised Superiority Clinical Trial of Azithromycin Versus Usual Care In Ambulatory COVID-19 (ATOMIC2) (ATOMIC2) | 04381962 | Y | AZM/ Std care | Oxford, UK | COVID | 800 | 11.05.2020 | Randomised |
|  | Hydroxychloroquine and Zinc With Either Azithromycin or Doxycycline for Treatment of COVID-19 in Outpatient Setting | 04370782 | Y | (HCQ + AZM + zinc sulfate) / (HCQ + doxycycline + zinc sulfate) | New York, USA | High risk COVID | 750 | 01.05.2020 | Randomised |
|  | Treatment for COVID-19 in High-Risk Adult Outpatients | 04354428 | Y | Placebo / HCQ / HCQ + AZM | USA | COVID + pneumonia + (1+risk factor) | 630 | 21.04.2020 | Randomised |
|  | Treatment in Patients With Suspected or Confirmed COVID-19 With Early Moderate or Severe Disease (RCT) | 04344444 | Y | HCQ / HCQ + AZM / supportive care | New Orleans, USA | Mild to moderate COVID | 600 | 14.04.2020 | Randomised |
|  | Pragmatic Factorial Trial of Hydroxychloroquine, Azithromycin, or Both for Treatment of Severe SARS-CoV-2 Infection | 04335552 | Y | HCQ + AZM / HCQ / AZM / Std of care | N Carolina, USA | COVID | 500 | 06.04.2020 | Randomised |
|  | Use of Hydroxychloroquine Alone or Associated for Inpatients With SARS-CoV2 Virus (COVID-19) | 04361461 | Y | HCQ / HCQ + AZM | Brasil | Moderate to severe COVID | 500 | 24.04.2020 | Randomised |
|  | Hydroxychloroquine Monotherapy and in Combination With Azithromycin in Patients With Moderate and Severe COVID-19 Disease | 04358081 | Y | HCQ / HCQ + AZM/ placebo | California, USA | Hospitalised for COVID | 444 | 22.04.2020 | Randomised |
|  | Safety and Efficacy of Hydroxychloroquine Associated With Azithromycin in SARS-CoV2 Virus (Coalition Covid-19 Brasil II) | 04321278 | Y | HCQ + AZM / HCQ | Brasil | Moderate to severe COVID | 440 | 25.03.2020 | Randomised |
| HAHPS | Hydroxychloroquine vs. Azithromycin for Hospitalized Patients With Suspected or Confirmed COVID-19 (HAHPS) | 04329832 | Y | AZM / HCQ | Utah, USA | Admitted for COVID | 300 | 01.04.2020 | Randomised |
| TOCOVID | Clinical Trial of Combined Use of Hydroxychloroquine, Azithromycin, and Tocilizumab for the Treatment of COVID-19 (TOCOVID) | 04332094 | Y | AZM + HCQ + tocilizumab / AZM + HCQ | Spain | Mild COVID | 276 | 02.04.2020 | Randomised |
| COSTA | Open Label Study to Compare Efficacy, Safety and Tolerability of Hydroxychloroquine Combined With Azithromycin Compared to Hydroxychloroquine Combined With Camostat Mesylate and to "no Treatment" in SARS CoV 2 Virus (COSTA) | 04355052 | Y | (AZM + HCQ)/(HCQ + camostat mesylate) | Israel | Mild COVID | 250 | 11.04.20 | Randomised |
| AZIQUINE-ICU | Azithromycin Added to Hydrochloroquine in Patients Admitted to Intensive Care With COVID-19: Randomised Controlled Trial (AZIQUINE-ICU) | 04339816 | Y | HCQ + AZM / HCQ + placebo / placebo + placebo | Czech Republic | ICU COVID | 240 | 09.04.2020 | Randomised |
|  | Novel Agents for Treatment of High-risk COVID-19 Positive Patients | 04374019 | Y | HCQ + HCQ / HCQ + AZM / HCQ + ivermectin /camostat mesilate | Kentucky, USA | COVID, high risk | 240 | 05.05.2020 | Randomised |
| PorPAC-COVID | Proactive Prophylaxis With Azithromycin and hydroxychloroquine in Hospitalized Patients With COVID-19 (ProPAC-COVID) | 04322396 | Y | AZM / HCQ / placebo | Denmark | Hospitalised for COVID | 226 | 26.03.2020 | Randomised |
|  | Randomized Comparison of Combination Azithromycin and Hydroxychloroquine vs. Hydroxychloroquine Alone for the Treatment of Confirmed COVID-19 | 04336332 | Y | AZM + HCQ / HCQ / delayed HCQ | New Jersey, USA | COVID | 160 | 07.04.2020 | Randomised |
| PROBIOZOVID | Oxygen-Ozone as Adjuvant Treatment in Early Control of COVID-19 Progression and Modulation of the Gut Microbial Flora (PROBIOZOVID) | 04366089 | Y | AZM +HCQ / oxygen ozone + probiotic | Italy | Hospitalised for COVID | 152 | 28.04.2020 | Randomised |
| COVIDOC | Hydroxychloroquine Plus Azithromycin Versus Hydroxychloroquine for COVID-19 Pneumonia (COVIDOC Trial) (COVIDOC) | 04345861 | Y | HCQ + placebo/ HCQ + AZM | France | Hospitalised for COVID + pneumonia | 150 | 15.04.2020 | Randomised |
| REP-COVID | Plasma Exchange in Patients With COVID-19 Disease and Invasive Mechanical Ventilation: a Randomized Controlled Trial (REP-COVID) | 04374539 | Y | Plasma exchange + polyclonal immunoglobulin + Std treatment [opinavir or ritonavir + HCQ +AZM + tocilizumab _ methylprednisolone + anakinra + clexane] / Std treatment | Barcelona | ICU COVID | 116 | 05.05.2020 | Randomised |
|  | Convalescent Plasma of Covid-19 to Treat SARS-COV-2 a Randomized Doble Blind 2 Center Trial (CPC-SARS) | 04405310 | Y | Hyperimmune Plasma from Convalescent patients + HCQ + AZM / HCQ + AZM +Hartman solution | Mexico | Moderate to severe COVID | 80 | 28.05.2020 | Randomised |
|  | The Effects of Standard Protocol With or Without Colchicine in Covid-19 Infection | 04360980 | Y | Colchicine + Std treatment [vit c+tiamine + selenium + omega 3 + vit a +vita d + AZM + ceftriazone + kaletra] / Std treatment | Iran | COVID | 80 | 24.04.2020 | Randomised |
| HyAzOUT | Hydroxychloroquine vs. Azithromycin for Outpatients in Utah With COVID-19 (HyAzOUT) | 04334382 | Y | AZM / HCQ | Utah, USA | >44 y outpatients, COVID-19 |  | 06.04.2020 | Randomised |
| TEACH-COVID | Evaluation of the efficacy of the hydroxychloroquine-Azithromycin combination in the prevention of COVID-19 related SDRA | 04347512 | With-drawn | HCQ + AZM / HCQ / control | France | COVID | 0 | 15.04.2020 | Randomised |
|  | Hydroxychloroquine,Hydroxychloroquine,Azithromycin in the Treatment of SARS CoV-2 Infection (WU352) | 04341727 | Suspended | HCQ / HCQ + AZM / chloroquine sulphate / chloroquine sulphate + AZM | USA | Hospitalised for COVID | 500 | 10.04.2020 | Randomised |
| CORIMUNO-VIRO | Study of Immune Modulatory Drugs and Other Treatments in COVID-19 Patients: Sarilumab, Azithromycin, Hydroxychloroquine Trial - CORIMUNO-19 - VIRO (CORIMUNO-VIRO) | 04341870 | Suspended | Sarilumab +AZM + HCQ / sarilumab only | France | COVID | 27 | 10.04.2020 | Randomised |
|  | The Fleming [FMTVDM] Directed CoVid-19 Treatment Protocol (FMTVDM) | 04349410 | Y | HCQ + AZM / HCQ + Doxycycline / HCQ + Clindamycin / HCQ + Clindamycin + Primaquine - low dose/ HCQ + Clindamycin +Primaquine - high dose. / Remdesivir / Tocilizumab / Methylprednisolone / Interferon-Alpha2B / Losartan /Convalescent Serum | USA | COVID | 500 | 16.04.2020 | Randomised |
|  | Efficacy of Ivermectin as Add on Therapy in COVID19 Patients | 04343092 | Completed | HCQ + AZM / HCQ + AZM + IVM | Iraq | COVID + pneumonia | 100 | 13.04.2020 | Randomised |
| ONCOVID | Epidemiology of SARS-CoV-2 and Mortality to Covid19 Disease in French Cancer Patient | 04341207 | Y | AZM + HCQ | France | >18 y with advanced malignancy | 1000 | 10.04.2020 | Non-randomised |
| COVID-RPC | Observational Cohort of COVID-19 Patients at Raymond-Poincare (COVID-RPC) | 04364698 | Y | Observational study of treatment effects of patients treated with HCQ+AZM | France | COVID | 500 | 28.04.2020 | Observational cohort study |
|  | Efficacy and Safety of Hydroxychloroquine and Azithromycin for the Treatment of Hospitalized Patients With Moderate to Severe COVID-19 | 04329572 | Suspended | AZM + HCQ | Brasil | Moderate to severe COVID | 400 | 01.04.2020 | Non-randomised |
|  | Effect of Treatments in Patients Hospitalized for Severe COVID-19 Pneumonia: a Multicenter Cohort Study | 04365764 | Y | Retrospective analysis of outcomes from treatments | France | Hospitalised for severe COVID | 400 | 28.04.2020 | Case control |
|  | Efficacy and Safety of Hydroxychloroquine and Azithromycin for the Treatment of Ambulatory Patients With Mild COVID-19 | 04348474 | Suspended | AZM + HCQ | Brasil | Mild ambulatory COVID-19 >70 y or <70 y with mild COVID + risk factors | 200 | 16.04.2020 | Non-randomised |
|  | Factors Associated With Clinical Outcomes in Patients Hospitalized for Covid-19 in GHT-93 Est | 04366206 | Y | Retrospective analysis from EPR of treatments. | France | Hospitalised for COVID | 143 | 28.04.2020 | Prospective cohort study |
|  | Low Dose Anti-inflammatory Radiotherapy for the Treatment of Pneumonia by COVID-19 | 04380818 | Y | Std care [HCQ + ritonvari-lopinavir + tocilizumab + AZM + corticosteroid +LMWH + oxygen]/ bilateral low dose lung irradiation + Std care | Spain | Moderate to severe COVID | 106 | 08.05.2020 | Non-randomised |
| PPT-COVID | Patient Preference Trial for COVID-19 (PPT-COVID) (PPT-COVID) | 04418193 | Y | HCQ / HCQ + AZM / Std care | Monaco | Not in a randomized trial, positive COVID, >75 y | 100 | 05.06.2020 | Non-randomised, observational |
|  | Evaluate the Efficacy and Safety of Oral Hydroxychloroquine, Indomethacin and Zithromax in Subjects With Mild Symptoms of COVID-19 (COVID-19) | 04344457 | Y | HCQ / indomethacin / AZM | Arizona, USA | Mild COVID | 80 | 14.04.2020 | Non-randomised |
|  | Ivermectin-Azithromycin-Cholecalciferol (IvAzCol) Combination Therapy for COVID-19 (IvAzCol) | 04399746 | Y | AZM + Ivermectin + cholecalciferol / no intervention | Mexico | Mild COVID | 30 | 22.05.2020 | Non-randomised |
|  | Atovaquone and Azithromycin Combination for Confirmed COVID-19 Infection | 04339426 | Y | AZM + atovaquone | Arizona, USA | COVID, high risk | 25 | 09.04.2020 | Non-randomised |
| ULTRA-COVID | Ultra Low Doses of Therapy With Radiation Applicated to COVID-19 (ULTRA-COVID) | 04394182 | Y | Ultra low dose radiotherapy +lopinavir-ritonavir + HCQ + AZM+ PipTaz + LMWH + corticosteroid + tocilizumab | Spain | Severe COVID + pneumonia | 15 | 19.05.2020 | N/A |
|  |  |  |  |  |  |  |  |  |  |
| Abbreviations: AZM, azithromycin; COVID, coronavirus-19 disease; CAP, community acquired pneumonia; HCQ, hydroxychloroquine; ICU, intensive care unit; IFN, interferon; IVM, ivermectin; LMWH, low molecular weight heparin; N/A, not applicable; NCT, National Clinical Trial number; vit, vitamin; Std, standard; TLR, Toll-like receptor; y, year. | | | | | | | | | |
